# GESTATIS: application for the execution and documentation of nurse-midwifery clinical assessment

**DOI:** 10.1590/0034-7167-2025-0374

**Published:** 2026-11-06

**Authors:** Heric Maia Gomes, Lorena Teixeira da Silva, Leticia Pontes, Mitzy Tannia Reichembach Danski

**Affiliations:** I Universidade Federal do Paraná. Curitiba, Paraná, Brazil

**Keywords:** Mobile Applications, Nursing Assessment, Obstetric Nursing, Nursing Process, Technology., Aplicaciones Móviles, Evaluación en Enfermería, Enfermería Obstétrica, Proceso de Enfermería, Tecnología.

## Abstract

**Objectives:**

to describe the development and content validity of a mobile application designed to optimize the Nursing Process in the nurse-midwifery context.

**Methods:**

a methodological study divided into three phases: exploratory phase; application development and validity; and prototype registration and availability.

**Results:**

an innovative technology capable of supporting the nurse-midwifery process through a mobile application was developed. GESTATIS uses logical algorithms based on truth tables for alerts, scale summation, and decision support in selecting the main nursing diagnoses. It compiles recorded data and generates a file in Portable Document Format. Content validity demonstrated relevance regarding the main nurse-midwifery nursing diagnoses, considered highly relevant: risk for unstable blood glucose level; impaired urinary elimination; risk for shock; risk for infection; and labor pain.

**Conclusions:**

GESTATIS is a flexible and replicable technology with the potential to improve care quality, strengthen nurses’ autonomy, and generate positive impacts in nurse-midwifery.

## RESUMEN

**Objetivos:**

describir el desarrollo y la validación de contenido de una aplicación móvil para optimizar el proceso de enfermería en el contexto obstétrico.

**Método:**

Estudio metodológico dividido en tres fases: exploratoria; desarrollo; registro.

**Resultados:**

se creó una tecnología innovadora capaz de asistir el proceso de enfermería obstétrica mediante una aplicación móvil. GESTATIS utiliza algoritmos lógicos en tablas de verdad para alertas, suma de escalas y apoyo a la selección de los principales diagnósticos de enfermería. Recopila los datos registrados y genera un archivo en formato PDF. La validación del contenido fue relevante para diagnósticos de enfermería obstétrica, considerados de alta pertinencia: riesgo de glucemia inestable; alteración de la eliminación urinaria; riesgo de shock; riesgo de infección; y dolor durante el parto.

**Conclusiones:**

GESTATIS es una tecnología flexible y replicable con potencial para mejorar la atención, fortalecer la autonomía de enfermería y generar impactos positivos en la enfermería obstétrica.

## INTRODUCTION

In recent years, Brazil has experienced significant growth in the adoption of digital technologies aimed at obstetric healthcare, especially through mobile applications, which enhance prenatal monitoring and communication between pregnant women and nursing teams^(1)^.

A recent study conducted with applications in the country demonstrated the effectiveness of monitoring gestational clinical conditions such as hypertension, diabetes, and risk of preterm birth, while also promoting self-care, adherence to consultations, and communication between professionals and patients. Expansion of these resources is integrated into the strengthening of digital health in Brazil, encompassing telemedicine, artificial intelligence, and the Internet of Things as strategic tools to expand access, efficiency, and safety in obstetric care^(2)^.

The increasing use of mobile applications and digital systems has proven effective in structuring nursing actions, enhancing patient safety, and improving documentation of clinical interventions. A recent study indicates that these applications contribute to error prevention, improvement of communication among teams, and automation of safety protocols^(3)^.

With the growing incorporation of mobile devices into society, especially smartphones, the healthcare sector has explored these resources as a means of improving quality of care. In recent studies in nursing and pre-hospital care, mobile applications have been recognized as effective tools for expanding access, integrating services, optimizing team response time, and promoting safety in clinical care. The use of applications to support decision-making, electronic documentation, and protocol automation has made care more effective, safer, and evidence-based^(4)^.

Within nursing, the Nursing Process (NP) stands out as an essential tool for ensuring quality of care. Resolution COFEN 736/2024 updates and standardizes implementation of the NP in five stages: nursing assessment; nursing diagnosis; planning; implementation; and assessment. In the nurse-midwifery context, its proper application is crucial for early identification of risks to pregnant women and fetuses, in addition to planning personalized interventions and systematically documenting clinical management^(5)^.

However, several challenges persist in implementation of the NP, especially in contexts characterized by high patient volume and shortage of professionals - factors that hinder its effective applicability. From this perspective, integration of health technologies through mobile devices, dashboards, and support software emerges as an innovative strategy to strengthen nurse-midwifery clinical practice, promoting greater safety, organization, agility in decision-making, and consequently superior quality in care delivery^(6)^.

During clinical practice in a maternal-child hospital unit in the interior of Rondônia, the absence of an instrument encompassing all stages of the NP in the nurse-midwifery context was identified. The printed instrument used showed low adherence among nurses working in the obstetric ward, resulting in incomplete documentation and deficient care planning. Therefore, the need emerged to develop a care technology in the form of a mobile application capable of supporting nurses in carrying out the NP in the nurse-midwifery context in a structured, efficient, and evidence-based manner^(7)^.

## OBJECTIVES

To describe the development process and content validity of a mobile application designed to optimize the NP in the nurse-midwifery context.

## METHODS

This is a methodological study with an applied approach aimed at development of assistive technological production, divided into three phases: exploratory phase, application development phase, and content validity phase. This study derives from the dissertation entitled “*GESTATIS: Aplicativo para Execução e Registro da Avaliação Clínica de Enfermagem Obstétrica*”.

Methodological research aims to produce knowledge to solve specific problems identified in practice. Widely used in nursing, it involves production, construction, validity, and assessment of instruments, focusing on development of new products^(8)^.

During the exploratory phase, an integrative literature review was conducted based on recommendations from the Preferred Reporting Items for Systematic Reviews and Meta-Analyses methodology and the PICO strategy, an acronym for Patient/Population, Intervention, Comparison, and Outcome, aiming to identify Brazilian publications regarding the NP in hospitalized pregnant women^(8)^.

The databases used were the Virtual Health Library, Latin American and Caribbean Health Sciences Literature, Nursing Database, Scientific Electronic Library Online, and Medical Literature Analysis and Retrieval System Online. Studies published between 2017 and 2022 with full texts available in Portuguese, English, and Spanish were included.

In the application development phase, content selection was conducted based on Ministry of Health protocols and nurse-midwives’ clinical practice. Data definition was guided by Wanda Horta’s Theory of Basic Human Needs. Subsequently, interface prototyping was performed using the Figma platform, followed by practical development using the Dart programming language and FlutterFlow framework, with backend structured in Firebase.

Content validity was performed through a questionnaire administered via the free Google Forms^®^ platform, comprising two items: (a) identification data, academic background, and professional experience; and (b) knowledge and professional practice regarding care for hospitalized pregnant women and assessment of the relevance level of the main nursing diagnoses selected by researchers according to NANDA-I (2021-2023)^(6)^. Data collection was conducted between November 2023 and January 2024, according to each participant’s availability. Of the 12 nurses from the obstetric ward of *Hospital Municipal Materno Infantil*, eight agreed to participate in the content validity process, three were on leave during data collection, and one declined participation.

For purposes of content analysis and interpretation of the obtained information, content analysis according to Laurence Bardin^(9)^ was used, an appropriate technique for exploring participant information collected during application of a semi-structured questionnaire. This proposal divides content analysis into three stages: pre-analysis; material exploration; and treatment of results, inference, and interpretation. The applied techniques underwent established stages to achieve construction of content within the research field^(9)^. Information analysis began with floating reading, formulation of hypotheses, and definition of objectives. After material exploration and treatment of results, findings were explored for content analysis related to nurse-midwifery assessment.

In the third phase, the application was registered with the Instituto Nacional da Propriedade Industrial under registration number BR512024001560-4 and made available for testing on Android and iPhone Operating System (iOS) devices.

## RESULTS

The GESTATIS application was developed and is subdivided into an interactive menu organized into tabs and sections according to the nursing assessment.

The application includes 13 sections: a user registration/login page; a control panel for organization; a professionals list (administrators); a patients list (users); a professionals list page; a patients list page; and an interactive menu containing information separated into topics related to nurse-midwifery assessment (hospital admission data, obstetric history, physical examination tab, psychosocial-spiritual tab, limbs tab, cardiorespiratory tab, gastro-genitourinary tab, and diagnosis tab). The application also provides spaces for entering complementary examination results and a numerical pain scale based on facial expressions.

The application includes decision algorithms such as: a function for calculating length of hospital stay and recording previous hospitalizations; an algorithm for calculating gestational age and estimated date of delivery based on insertion of a reference date; an algorithm for calculating Body Mass Index according to patient weight and height; a data structure for displaying and comparing results from the most recent examination with previous ones; color and text alerts for vital signs, including indexation of uterine height according to gestational age; an algorithm for selecting, in cases of absent spontaneous ventilation, Venturi masks and nasal catheters associated with their respective oxygen flow rates; an emergency alert related to cervical measurement; a decision-structure algorithm based on Boolean logic for definition of probable nurse-midwifery nursing diagnoses; and an algorithm for generating, visualizing, saving, sharing, and printing Portable Document Format (PDF) nursing progress notes based on patient data and professional confirmation of diagnoses generated by GESTATIS.

During nurse-midwifery assessment, as shown in Figure 1, the application also offers suggestions regarding the main nursing diagnoses according to alterations identified in obstetric physical examination, based on NANDA-I (2021-2023). After assessment, a native PDF document is generated presenting the identified alterations.

**Figure 1:**
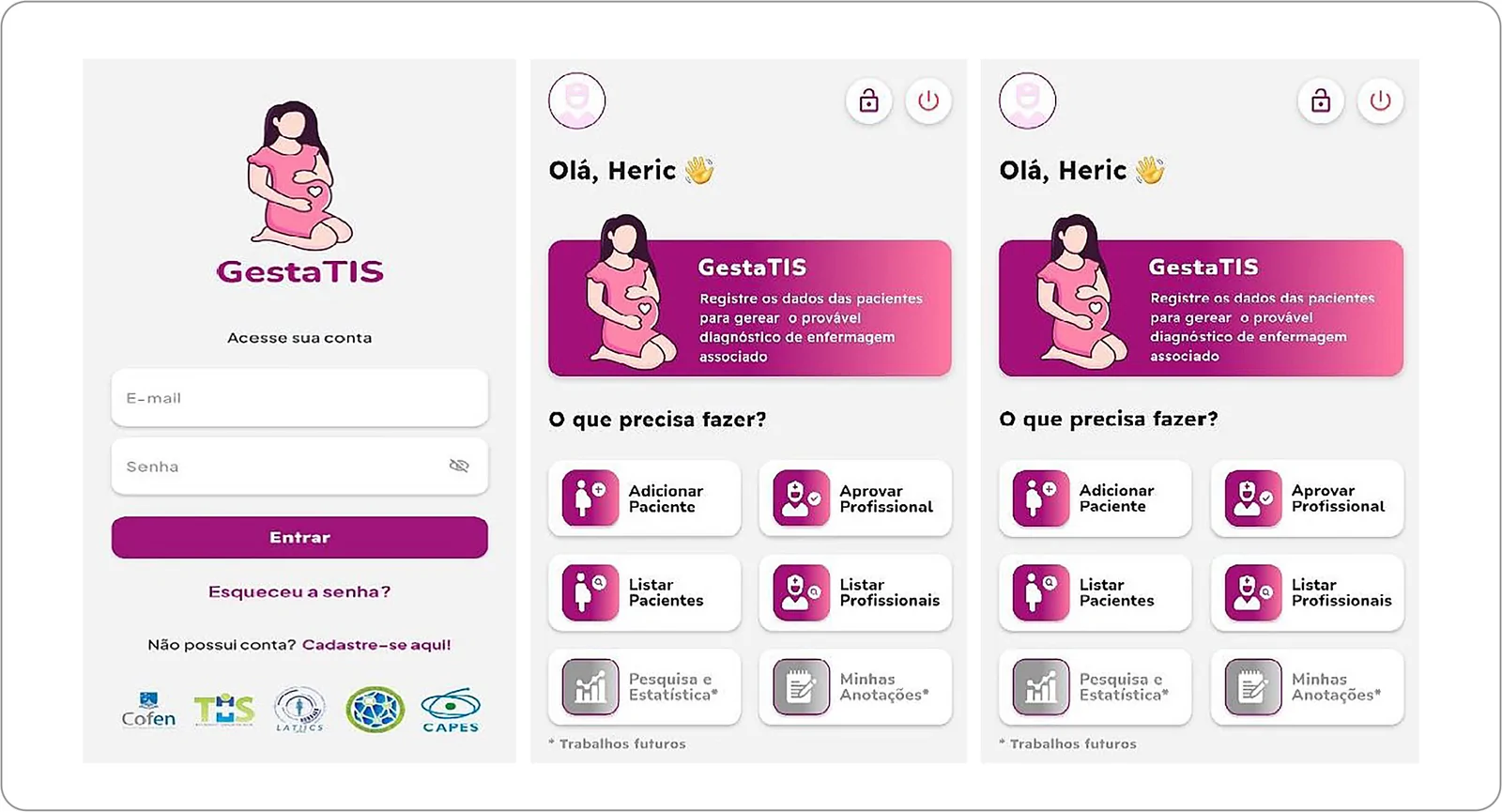
GESTATIS application interfaces, Curitiba, Paraná, Brazil, 2024

### Results of the content assessment of nurse-midwifery practice

Content assessment was conducted with the objective of verifying the relevance of the main nursing diagnoses in obstetric practice according to the NANDA-I (2021-2023) classification. For this purpose, a semi-structured questionnaire developed on the Google Forms^®^ platform was applied between November 2023 and January 2024. The instrument addressed two main domains: (a) professional identification, academic background, and professional experience; and (b) assessment of knowledge and clinical practice in the care of hospitalized pregnant women, emphasizing the relevance of 26 nursing diagnoses selected by the researchers. The questionnaire contained 17 questions, of which 11 were open-ended and six were closed-ended. Assessment of diagnoses was based on a five-level scale: not relevant; slightly relevant; somewhat relevant; very relevant; and extremely relevant.

Among the 26 assessed diagnoses, eight stood out with the highest frequency of assessment as “very” or “extremely relevant”, demonstrating strong applicability in obstetric care according to study participants, as presented in Table 1.

**Table 1:** Relevant classified nursing diagnoses in the obstetric assessment of pregnant women

Nursing diagnosis	High relevance
• Risk for unstable blood glucose	62.5%
• Anxiety	62.5%
• Obesity	50%
• Impaired urinary elimination	50%
• Ineffective breathing pattern	50%
• Risk for shock	50%
• Labor pain	50%
• Nausea	50%

These findings demonstrate the centrality of pregnant women’s clinical and psychosocial aspects during nurse-midwifery assessment.

## DISCUSSION

The application operates through Android and iOS platforms. With an active internet connection and after downloading the application onto a mobile device, users must request permission from the administrator, who, after assessment, may authorize access to the platform. Once permission is granted, nurses may navigate in a structured manner, registering patients to be assessed and entering data collected during anamnesis and physical examination.

Based on alterations identified during obstetric assessment, the system itself automatically suggests the main nursing diagnoses according to clinical findings. It is the nurse’s responsibility, through clinical reasoning, to validate or adjust these suggestions and make the most appropriate decision for each case.

All inserted data related to anamnesis, physical examination, and nursing diagnoses are automatically organized and exported in PDF format. This document may be attached to the patient’s medical record and also allows inclusion of nursing interventions to be defined according to management established in the NP.

Regarding relevant aspects in obstetric assessment, essential elements for clinical practice were mentioned: 50% of participants highlighted obstetric history as fundamental in assessment; 25% reported vaginal discharge and bleeding as relevant clinical findings; and 25% emphasized emotional factors such as fear and anxiety during the prepartum period.

There were no specific mentions regarding family or spiritual needs within the hospital context. Regarding the content analysis procedure, Bardin’s analysis^(2)^ was used, structured into three main stages: pre-analysis - floating reading of data and definition of objectives; material exploration - categorization and coding of reports; and treatment of results and interpretation - critical interpretation based on constructed categories.

Information was organized into categories and subcategories: psychobiological - level of consciousness, pain, sleep, nutrition, elimination, mobility, and ventilation; psychosocial - emotional state, psychological follow-up, and family support; and psychospiritual - spiritual assessment.

The proposed innovation consists of application of logical functions and algorithmic combinations organized into truth tables capable of integrating clinical data, performing automated scale summation, and issuing alerts in response to alterations in vital or obstetric parameters. This technological approach, designed for mobile devices, aims to qualify and modernize implementation of the NP, especially in the nurse-midwifery context, through an agile, secure, and responsive interface. In addition to increasing efficiency and accuracy of clinical assessments, the solution contributes to reducing the use of printed forms, preventing waste and optimizing time and resources used by the nursing team within hospital settings.

### Study limitations

As a limitation of the study, the absence of application assessment within the expected timeframe and the high level of complexity involved in its development stand out. These factors contributed to difficulties in monitoring established goals and complying with the initially proposed schedule.

### Contributions to nursing

Given the innovative potential of using mobile devices in nursing care, GESTATIS is a technology that can be easily replicated in different clinical and hospital units, with relevant social and economic impact and significant contribution to qualification of care delivery.

Its innovation lies in incorporation of logical functions and combinations of algorithms structured in truth tables, allowing, for example, automated summation of scales and issuance of alerts in response to alterations in vital records or obstetric parameters. This technological approach contributes to faster, safer, and data-based decision-making, optimizing the NP in the nurse-midwifery context.

The application is available on Android and Apple operating systems. In the application store, users should search for “gesta-tis”.

## CONCLUSIONS

The developed application fully encompasses aspects related to nurse-midwifery assessment, considering patients’ psychobiological, psychosocial, and psychospiritual needs. Based on data collected during assessment, the system automatically suggests the main nursing diagnoses aligned with evidence-based clinical practice.

GESTATIS proposes a new concept for obstetric assessment by using mobile software as a support tool, promoting greater agility, organization, and effectiveness in implementation of the NP in different obstetric care settings. The application contributes to practical implementation of the NP in a facilitated, systematized, and safe manner.

This is a flexible technology open to incorporation of new resources, such as artificial intelligence mechanisms aimed at identification of clinical patterns (signs, symptoms, and behaviors), in addition to intelligent communication solutions such as integration with tools based on natural language, such as ChatGPT.

It is concluded that GESTATIS represents a significant innovation by bringing together, in a technological solution with replication potential throughout the national territory, the main elements of the NP in the nurse-midwifery context, validated in terms of content. The tool has high potential to generate positive impacts on quality of care, optimization of healthcare services, and social and economic indicators of nursing in Brazil.

## Data Availability

The research data are available within the article.
