# Supplementary material for: GESTATIS: application for the execution and documentation of nurse-midwifery clinical assessment
Source: Rev Bras Enferm. 2026 Nov 6;79:e20250374. doi: 10.1590/0034-7167-2025-0374 (PMC13743048; doi:10.1590/0034-7167-2025-0374)
Supplement: Translation

# Translation: GESTATIS: aplicativo para execução e registro da avaliação clínica do enfermeiro obstetra

**DOI:** 10.1590/0034-7167-2025-0374pt

**Authors:** Heric Maia Gomes, Lorena Teixeira da Silva, Leticia Pontes, Mitzy Tannia Reichembach Danski

**Affiliations:** I Universidade Federal do Paraná. Curitiba, Paraná, Brasil

**Keywords:** Aplicativos Móveis, Avaliação em Enfermagem, Enfermagem Obstétrica, Processo de Enfermagem, Tecnologia.

## Abstract

**Objetivos:**

descrever o desenvolvimento e a validação de conteúdo de um aplicativo móvel para otimizar o processo de enfermagem no contexto obstétrico.

**Métodos:**

estudo metodológico, dividido em três fases: exploratória; desenvolvimento e validação do aplicativo; e registro e disponibilização do protótipo.

**Resultados:**

criou-se tecnologia inovadora capaz de auxiliar o processo de enfermagem obstétrica via aplicativo móvel. O GESTATIS utiliza algoritmos lógicos em tabelas-verdade para alertas, somatório de escalas e apoio à decisão para escolha dos principais diagnósticos de enfermagem. Ele compila os dados registrados e gera arquivo em *Portable Document Format*. A validação de conteúdo foi considerada muitíssimo pertinente aos principais diagnósticos de enfermagem obstétrica: risco de glicemia instável; eliminação urinária prejudicada; risco de choque; risco de infecção; e dor no trabalho de parto.

**Conclusões:**

o GESTATIS é uma tecnologia flexível, replicável, com potencial para qualificar a assistência, fortalecer a autonomia do enfermeiro e gerar impactos positivos na enfermagem obstétrica.

## INTRODUÇÃO

Nos últimos anos, o Brasil tem observado um crescimento expressivo na adoção de tecnologias digitais voltadas à saúde obstétrica, especialmente por meio de aplicativos móveis, que potencializam o monitoramento pré-natal e a comunicação entre gestantes e equipes de enfermagem^(1)^.

Estudo recente realizado com aplicativos no país demonstra a eficácia do acompanhamento de condições clínicas gestacionais, como hipertensão, diabetes e risco de parto prematuro, e também favorece o autocuidado, a adesão às consultas e a comunicação entre profissionais e pacientes. A ampliação desses recursos integra-se ao fortalecimento da saúde digital no Brasil, que abrange a telemedicina, a inteligência artificial e a Internet das Coisas como ferramentas estratégicas para ampliar o acesso, a eficiência e a segurança da assistência obstétrica^(2)^.

O uso crescente de aplicativos móveis e sistemas digitais tem se mostrado eficaz na estruturação das ações de enfermagem, ampliando a segurança do paciente e aprimorando o registro das intervenções clínicas. Estudo recente indica que esses apps contribuem para a prevenção de erros, a melhoria da comunicação entre equipes e a automação de protocolos de segurança^(3)^.

Com a crescente inserção dos dispositivos móveis na sociedade, especialmente dos *smartphones*, o setor da saúde tem explorado esses recursos como meio de aprimorar a qualidade da assistência. Em estudos recentes na área da enfermagem e do atendimento pré-hospitalar, os aplicativos móveis vêm sendo reconhecidos como instrumentos eficazes para ampliar o acesso, integrar serviços, otimizar o tempo de resposta das equipes e promover a segurança no cuidado clínico. A utilização de aplicativos para apoio à tomada de decisão, ao registro eletrônico e à automação de protocolos tornou o cuidado mais eficaz, seguro e fundamentado em evidências^(4)^.

No âmbito da enfermagem, destaca-se o Processo de Enfermagem (PE) como ferramenta essencial para garantir a qualidade da assistência. A Resolução COFEN nº 736/2024 atualiza e normatiza a aplicação do PE em cinco etapas: avaliação de enfermagem; diagnóstico de enfermagem; planejamento; implementação; e evolução. No contexto obstétrico, a sua aplicação adequada é crucial para identificar precocemente riscos à gestante e ao feto, além de planejar intervenções personalizadas e registrar as condutas de forma sistemática^(5)^.

Entretanto, persistem diversos desafios na implementação do PE, especialmente em contextos de alto volume de atendimentos e escassez de profissionais - fatores que dificultam a sua aplicabilidade efetiva. Nessa perspectiva, a integração de tecnologias em saúde por meio de dispositivos móveis, *dashboards* e *softwares* de apoio surge como uma estratégia inovadora para fortalecer a prática clínica obstétrica, promovendo maior segurança, organização, agilidade na tomada de decisão e, consequentemente, qualidade superior na assistência prestada^(6)^.

Durante a prática assistencial em uma unidade hospitalar materno-infantil no interior de Rondônia, identificou-se a inexistência de um instrumento que contemplasse todas as etapas do PE no contexto obstétrico. O instrumento impresso utilizado era pouco aderido pelos enfermeiros da ala obstétrica, resultando em registros incompletos e no planejamento do cuidado deficitário. Diante disso, surgiu a necessidade de desenvolver uma tecnologia assistencial em forma de aplicativo móvel que pudesse ser utilizado como suporte ao enfermeiro na realização do PE no contexto obstétrico, de forma estruturada, eficiente e baseada em evidências^(7)^.

## OBJETIVOS

Descrever o processo de desenvolvimento e validação de conteúdo de um aplicativo móvel para otimizar o PE no contexto obstétrico.

## MÉTODO

Trata-se de estudo metodológico com abordagem aplicada, voltado à produção tecnológica assistencial, dividido em três fases: fase exploratória, fase de desenvolvimento do aplicativo e validação de conteúdo. Este estudo é derivado da dissertação intitulada “GESTATIS: Aplicativo para Execução e Registro da Avaliação Clínica de Enfermagem Obstétrica”.

A pesquisa metodológica tem como objetivo produzir conhecimento para solucionar problemas específicos, diagnosticados na prática. Amplamente utilizada pela enfermagem, ela envolve a produção, a construção, a validação e a avaliação de instrumentos, com foco no desenvolvimento de novos produtos^(8)^.

Na fase exploratória, foi realizada uma revisão integrativa da literatura com base nas recomendações da metodologia *Preferred Reporting Items for Systematic Reviews and Meta-Analyses* e na estratégia PICO, acrônimo para Paciente/População, Intervenção, Comparação e *Outcome* (desfecho), buscando identificar publicações brasileiras sobre o PE em gestantes hospitalizadas^(8)^.

As bases utilizadas foram a Biblioteca Virtual em Saúde, a Literatura Latino-Americana e do Caribe em Ciências da Saúde, a Base de Dados em enfermagem, a *Scientific Electronic Library Online* e a *Medical Literature Analysis and Retrieval System Online*. Incluíram-se estudos publicados entre 2017 e 2022, com textos completos disponíveis em português, inglês e espanhol.

Na fase de desenvolvimento do aplicativo, foi realizada a seleção do conteúdo com base em protocolos do Ministério da Saúde e na prática clínica de enfermeiros obstetras. A definição dos dados foi orientada pela Teoria das Necessidades Humanas Básicas de Wanda Horta. Posteriormente, foi realizada a prototipagem das interfaces utilizando a plataforma *Figma*, seguida do desenvolvimento prático com o uso da linguagem *Dart* e do *framework FlutterFlow*, com *backend* estruturado em *Firebase*.

A validação de conteúdo foi feita por meio de um questionário, através da plataforma gratuita *Google Forms^®^*, contemplando dois itens: a) dados de identificação, formação acadêmica e experiência profissional; b) conhecimento e prática profissional no atendimento de gestantes em hospital e avaliação quanto ao nível de pertinência dos principais diagnósticos de enfermagem selecionados entre os pesquisadores, conforme a NANDA-I (2021-2023)^(6)^. A coleta das informações foi realizada entre novembro de 2023 e janeiro de 2024, conforme a disponibilidade de cada participante. Do total de 12 enfermeiros da ala obstétrica do Hospital Municipal Materno Infantil, oito aceitaram participar do processo de validação de conteúdo, três estavam afastados durante a coleta de dados, e um não concordou em participar do estudo.

Para fins de análise de conteúdo e interpretação das informações obtidas, foi utilizada a análise de conteúdo segundo Bardin^(9)^, técnica adequada para exploração de informações dos participantes da pesquisa durante a aplicação do questionário semiestruturado. Essa proposta divide a análise de conteúdo em três etapas: Pré-análise; exploração do material; e tratamento dos resultados, inferência e interpretação. As técnicas aplicadas passaram por etapas estabelecidas para se chegar à construção do conteúdo no campo de pesquisa^(9)^. As informações foram analisadas com início em leitura flutuante, formulação de hipóteses e objetivos. Após a exploração do material e tratamento dos resultados, houve a exploração dos resultados para análise de conteúdo relacionado à avaliação de enfermagem obstétrica.

Na terceira fase, foi realizado o registro do aplicativo no Instituto Nacional da Propriedade Industrial, sob o número BR512024001560-4, e disponibilização para testes em dispositivos *Android* e *Iphone Operating System* (iOS).

## RESULTADOS

Foi desenvolvido o aplicativo GESTATIS, que é subdividido em menu interativo, organizado em abas e seções conforme avaliação de enfermagem.

O aplicativo possui 13 seções: uma página de cadastro/acesso do usuário; um painel de controle para organização; uma lista de profissionais (administradores); uma lista de pacientes (usuários); uma página de lista de profissionais; uma página de lista de pacientes; e um menu interativo com informações separadas por tópicos da avaliação de enfermagem obstétrica (dados de internação, antecedentes obstétricos, aba exame físico, aba psicossocioespiritual, membros, aba cardiorrespiratório, aba gastrogeniturinário e aba diagnóstico). Há também, disponível no aplicativo, espaços para preenchimento de resultados de exames complementares e escala numérica de dor por expressões faciais.

O aplicativo dispõe de algoritmos de decisões como: função para cálculo de dias de internação e registro de internações anteriores; algoritmo para cálculo de idade gestacional e provável data de parto a partir da inserção de uma data de referência; algoritmo para cálculo de Índice de Massa Corporal conforme peso e altura da paciente; estrutura de dados para exibir e comparar resultados do último exame com o anterior; alertas de cores e textos para os sinais vitais, incluindo a indexação da altura uterina à idade gestacional; algoritmo para selecionar, em caso de não ventilação espontânea, máscara de Venturi e cateter nasal, relacionados aos respectivos fluxos de oxigênio; alerta de urgência relacionado à medida do colo uterino; algoritmo de estrutura de decisão baseado em lógica booleana para definição de prováveis diagnósticos de enfermagem obstétrica; algoritmo para geração, visualização, salvamento, compartilhamento e impressão de arquivo em formato *Portable Document Format* (PDF) de evolução de enfermagem baseado nos dados da paciente e corroboração do profissional com os diagnósticos gerados pelo GESTATIS.

Durante a realização da avaliação obstétrica, conforme demonstrado na Figura 1, o aplicativo também oferece sugestões dos principais diagnósticos de enfermagem, conforme alterações no exame físico obstétrico, de acordo com a NANDA-I (2021-2023). Após a avaliação, é gerado um documento nativo em PDF com a apresentação das alterações encontradas.

**Figura 1:** Interfaces do aplicativo GESTATIS, Curitiba, Paraná, Brasil, 2024

### Resultados da avaliação de conteúdo da prática de enfermagem obstétrica

A avaliação de conteúdo foi conduzida com o objetivo de verificar a pertinência dos principais diagnósticos de enfermagem na prática obstétrica, conforme a classificação da NANDA-I (2021-2023). Para tanto, utilizou-se um questionário semiestruturado, desenvolvido na plataforma *Google Forms^®^*, aplicado entre novembro de 2023 e janeiro de 2024. O instrumento abordou dois principais eixos: (a) identificação profissional, formação acadêmica e experiência; e (b) avaliação do conhecimento e prática clínica no atendimento a gestantes em ambiente hospitalar, com ênfase na pertinência de 26 diagnósticos de enfermagem selecionados pelos pesquisadores. O questionário continha 17 questões, das quais 11 eram abertas e seis eram fechadas. A avaliação dos diagnósticos foi feita com base em uma escala de cinco níveis: nada pertinente; muito pouco pertinente; de algum modo pertinente; muito pertinente; e muitíssimo pertinente.

Dos 26 diagnósticos avaliados, destacaram-se oito com maior frequência de avaliação como “muito” ou “muitíssimo pertinente”, demonstrando forte aplicabilidade na assistência obstétrica segundo os participantes da pesquisa, conforme apresentado na Tabela 1.

**Tabela 1:** Diagnósticos de enfermagem classificados relevantes na avaliação obstétrica de gestantes

Diagnóstico de enfermagem	Alta pertinência
• Risco de glicemia instável	62,5%
• Ansiedade	62,5%
• Obesidade	50%
• Eliminação urinária prejudicada	50%
• Padrão respiratório ineficaz	50%
• Risco de choque	50%
• Dor no trabalho de parto	50%
• Náusea	50%

Esses dados demonstram a centralidade dos aspectos clínicos e psicossociais da gestante durante a avaliação de enfermagem obstétrica.

## DISCUSSÃO

O aplicativo funciona através das plataformas Android e iOS. Com uma conexão de internet ativa e após ter realizado o *download* do aplicativo em seu dispositivo móvel, o usuário deverá solicitar permissão ao administrador, que, após análise, poderá autorizar o acesso à plataforma. Uma vez concedida a permissão, o enfermeiro poderá navegar de forma estruturada, realizando o cadastro das pacientes a serem avaliadas e preenchendo os dados coletados durante a anamnese e o exame físico.

Com base nas alterações identificadas na avaliação obstétrica, o próprio sistema sugere automaticamente os principais diagnósticos de enfermagem, de acordo com os achados clínicos. Caberá ao enfermeiro, por meio do raciocínio clínico, validar ou ajustar essas sugestões e tomar a decisão mais adequada para cada caso.

Todos os dados inseridos referentes à anamnese, ao exame físico e aos diagnósticos de enfermagem serão organizados automaticamente e exportados em formato PDF. Esse documento poderá ser anexado ao prontuário da paciente, permitindo ainda a inclusão das intervenções de enfermagem a serem definidas conforme a conduta estabelecida no PE.

Quanto aos aspectos relevantes na avaliação obstétrica, foram mencionados elementos essenciais à prática assistencial: 50% dos participantes destacaram os antecedentes obstétricos como fundamentais na avaliação; 25% relataram perdas vaginais e sangramentos como achados clínicos relevantes; e 25% enfatizaram a presença de fatores emocionais, como medo e ansiedade no período pré-parto.

Não houve menções específicas às necessidades familiares ou espirituais no contexto hospitalar. Quanto ao procedimento de análise de conteúdo, utilizou-se a análise de acordo com Bardin^(2)^, estruturada em três etapas principais: pré-análise - leitura flutuante dos dados e definição dos objetivos; exploração do material - categorização e codificação dos relatos; e tratamento dos resultados e interpretação - interpretação crítica a partir das categorias construídas.

As informações foram organizadas em categorias e subcategorias: psicobiológica - nível de consciência, dor, sono, nutrição, eliminação, mobilidade e ventilação; psicossocial - estado emocional, acompanhamento psicológico e retaguarda familiar; e psicoespiritual - avaliação espiritual.

A proposta de inovação consiste na aplicação de funções lógicas e combinações algorítmicas organizadas em tabelas-verdade, que são capazes de integrar dados clínicos, realizar somas automatizadas de escalas e emitir alertas diante de alterações em parâmetros vitais ou obstétricos. Essa abordagem tecnológica, voltada para dispositivos móveis, tem como objetivo qualificar e modernizar a execução do PE, especialmente no contexto obstétrico, por meio de uma interface ágil, segura e responsiva. Além de ampliar a eficiência e a precisão das avaliações clínicas, a solução contribui para a redução do uso de formulários impressos, evitando desperdícios e otimizando o tempo e os recursos da equipe de enfermagem no ambiente hospitalar.

### Limitações do estudo

Como limitação do estudo, destaca-se a ausência de avaliação do aplicativo dentro do prazo previsto, além do nível elevado de complexidade envolvido em sua construção. Esses fatores contribuíram para dificuldades no acompanhamento das metas estabelecidas e no cumprimento do cronograma inicialmente proposto.

### Contribuições para a área

Diante do potencial inovador do uso de dispositivos móveis na assistência de enfermagem, o GESTATIS é uma tecnologia de fácil replicação em diferentes unidades clínicas e hospitalares, com relevante impacto social, econômico e na qualificação do cuidado prestado.

Sua inovação reside na incorporação de funções lógicas e combinações de algoritmos estruturados em tabelas-verdade, permitindo, por exemplo, o somatório automatizado de escalas e a emissão de alertas diante de alterações em registros vitais ou parâmetros obstétricos. Essa abordagem tecnológica contribui para uma tomada de decisão mais ágil, segura e baseada em dados, otimizando o PE no contexto obstétrico.

Está disponível no sistema operacional das plataformas Android e iOS. Na loja, deve-se buscar por “gesta-tis”.

## CONCLUSÕES

O aplicativo desenvolvido contempla integralmente os aspectos da avaliação de enfermagem obstétrica, considerando as necessidades psicobiológicas, psicossociais e psicoespirituais da paciente. Com base nos dados coletados durante a avaliação, o sistema sugere automaticamente os principais diagnósticos de enfermagem, alinhados à prática clínica baseada em evidências.

O GESTATIS propõe um novo conceito para a avaliação obstétrica ao utilizar um *software* móvel como ferramenta de apoio, promovendo maior agilidade, organização e efetividade na execução do PE em diferentes cenários da atenção obstétrica. O aplicativo contribui para a aplicação prática do PE de forma facilitada, sistematizada e segura.

Trata-se de uma tecnologia flexível e aberta à incorporação de novos recursos, como mecanismos de inteligência artificial voltados à identificação de padrões clínicos (sinais, sintomas e comportamentos), além de soluções em comunicação inteligente, como a integração com ferramentas baseadas em linguagem natural, a exemplo do ChatGPT.

Conclui-se que o GESTATIS representa uma inovação significativa ao reunir, em uma solução tecnológica com potencial de replicação em todo o território nacional, os principais elementos do PE no contexto obstétrico, validados em seu conteúdo. A ferramenta possui elevado potencial para gerar impactos positivos na qualidade da assistência, na otimização dos serviços de saúde e nos indicadores sociais e econômicos da enfermagem no Brasil.

## Data Availability

Os dados de pesquisa estão disponíveis no corpo do artigo.
